# ATP-induced autophagy is associated with rapid killing of intracellular mycobacteria within human monocytes/macrophages

**DOI:** 10.1186/1471-2172-9-35

**Published:** 2008-07-15

**Authors:** Debasis Biswas, Omar S Qureshi, Wing-Yiu Lee, Joanne E Croudace, Manuela Mura, David A Lammas

**Affiliations:** 1 Division of Immunity and Infection, The Medical School, University of Birmingham, B15 2TT, UK

## Abstract

**Background:**

We have previously reported that ATP treatment of *M bovis*-BCG infected human macrophages induces P2X_7 _receptor-dependent killing of intracellular mycobacteria. The mechanism mediating this bactericidal effect has not been full characterized but is known to be Ca^2+^-dependent and to promote the maturation and acidification of mycobacteria-containing phagosomes. In this study we demonstrate that the ATP/P2X_7_-mediated, mycobactericidal effect also involves the induction of cell autophagy.

**Results:**

We report that 3 mM ATP induces rapid cell autophagy in THP1 cells and monocyte-derived macrophages within 30 minutes post-treatment, as revealed by the expression of LC3-II bands on western blot analysis. Using Ca^2+^-free media and selective P2X_7 _agonists and antagonists, ATP-induced cell autophagy was shown to be Ca^2+ ^and P2X_7 _receptor-dependent. Electron microscopy of ATP-treated, BCG-infected MDMs revealed the presence of the bacteria within characteristic double-membraned autophagosomes. Confocal analysis further confirmed that pharmacological inhibition of autophagy by wortmannin or pre-treatment of macrophages with anti-P2X_7 _antibody blocked ATP-induced phago-lysosomal fusion. Induction of cell autophagy with ATP was also temporally associated with a fall in intracellular mycobacterial viability, which was suppressed by treatment with wortmannin or the selective P2X_7 _antagonist, oxidized ATP (oATP).

**Conclusion:**

We provide the first evidence that ATP/P2X_7_-mediated killing of intracellular mycobacteria involves the induction of cell autophagy. The findings support the hypothesis that autophagy plays a key role in the control of mycobacterial infections.

## Background

Tuberculosis continues to be a leading cause of human mortality and morbidity, with WHO figures estimating a global prevalence exceeding 14 million and mortality of approximately 1.6 million in the year 2005 [1]. The continuing global crisis has become further complicated by the emergence of multi-drug resistant strains of the bacteria and an increasing population of HIV-infected patients around the world. In view of this escalating clinical challenge, there is a growing need to develop novel therapeutic alternatives employing potent mycobactericidal mechanisms. Hence, there has been a lot of interest in cell autophagy as a potential immune defense mechanism against a number of bacterial pathogens, including mycobacteria [2-5]. Physiological or pharmacological induction of autophagy via cell starvation or treatment with rapamycin, have been reported to suppress mycobacterial survival within RAW cells through increased acidification and maturation of mycobacterial phagosomes [4].

It has been previously reported from our laboratory that treatment of infected human macrophages with adenosine 5'-triphosphate (ATP) was capable of killing mycobacteria by subverting the mycobacterium-induced block in phago-lysosomal fusion [6,7]. In the light of recent findings, the present study was undertaken to look for evidence of induction of cell autophagy within ATP-treated macrophages and of a role for this process in mediating its associated mycobactericidal activity. In this work we demonstrate, for the first time, that ATP induces autophagy in human macrophages within 30 minutes of exposure, which was associated with a subsequent decrease in *Mycobacterium bovis *BCG viability within infected cells. We further confirmed a role for extracellular Ca^2+ ^and delineated the identity of the purinergic receptor involved in this process by using Ca^2+^-free media and selective purinergic receptor agonists and antagonists. We were thus able to confirm that ATP induces rapid cell autophagy and killing of intracellular mycobacteria within infected human macrophages via a Ca^2+^-dependent process mediated by activation of P2X_7 _receptors. The results support the importance of autophagy as a mycobactericidal mechanism utilized by human macrophages and provide a possible rationale for the recently reported P2X_7 _polymorphisms associated with extra-pulmonary TB [8]. The findings also suggest a potential therapeutic role for P2X_7 _agonists in treating mycobacterial infections.

## Results

### ATP induces autophagy

To determine whether ATP was capable of inducing autophagy in THP1 cells and monocyte-derived macrophages (MDMs), the expression of the microtubule-associated (LC3-I) and membrane associated (LC3-II) forms of LC3 protein were assessed in both ATP-treated and untreated cells by Western Blot and Confocal microscopy.

**Figure 1:**
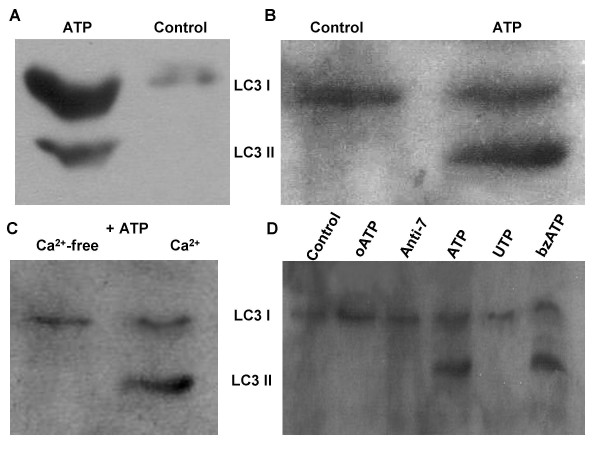
**Induction of autophagy in THP1 cells and MDMs, following treatment with ATP, is both calcium and P2X_7 _– dependent**. A. THP1 cells undergo autophagy (lane 1) following 30 minutes exposure to ATP (3 mM), as revealed by the presence of LC3-II (16 kDa) by Western blot. Lane 2 represents untreated control THP-1 cells. B. ATP (3 mM) treated MDMs also express LC3-II (lane 2), which is absent in non-treated control cells (lane 1). C. THP1 cells treated with 3 mM ATP for 30 minutes cultured in calcium-free medium (lane 1) and calcium-replete medium (lane 2). LC3-II expression was observed in ATP-treated cells cultured in calcium-replete medium (lane 2) but was absent in cells cultured in calcium-free media (lane 1). D. THP1 cells were treated with different P2 agonists and antagonists. Lane 1 represents untreated control cells, while lanes 2 and 3 represent cells pre-treated with oATP (0.3 mM) and anti-P2X_7 _antibody (3 μg/ml) for 2 hours and 1 hour respectively, prior to treatment with ATP (3 mM for 30 minutes). Lanes 4, 5 and 6 represent cells treated with ATP (3 mM), UTP (3 mM) and bzATP (3 mM) for 30 minutes. The presence of an LC3-II band, indicating cell autophagy, was observed only in lanes 4 and 6.

The processing of the 18 kDa microtubule-associated form of LC3 protein (LC3-I) to its 16 kDa, truncated form (LC3-II), is accepted as one of the standard read-outs of cell autophagy [9,10]. In Western blot studies, we observed expression of both LC3-I and LC3-II forms of LC3 protein in ATP-treated THP1 cells (Figure 1A) and MDMs, (Figure 1B) as revealed by bands with electrophoretic mobility corresponding to molecular masses of 18 kDa and 16 kDa respectively. In contrast, only the 18 kDa LC3-I band was expressed in control non-ATP treated THP1 or MDM cells (Figures 1A and 1B).

**Figure 2:**
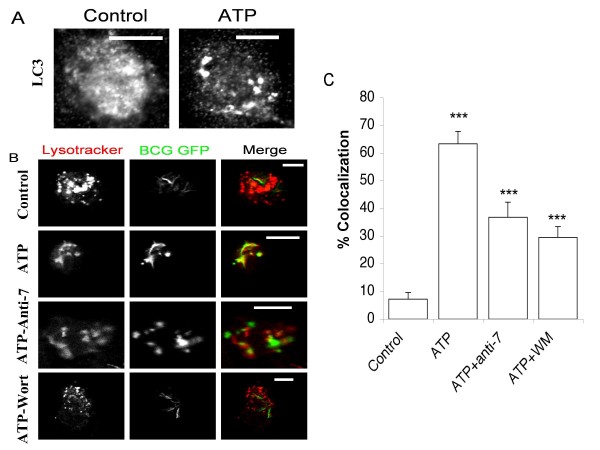
**ATP-induced autophagy triggers phagosome-lysosome fusion**. A. Confocal images of MDMs treated with 3 mM ATP for 30 minutes and immuno stained for intracellular LC3. B. Confocal images of live MDMs infected with GFP-BCG (green) and pre-pulsed with Lysotracker (red) to stain acidic lysosomes. Cells were pre-incubated with anti-P2X7 (3 μg/ml) or wortmannin (100 nM) for 1 hr and then treated with 3 mM ATP for 30 minutes. Note scale bars = 10 um. C. Graph showing the percentage of Lysotracker positive, GFP-BCG containing phagosomes. Histogram shows means ± s.e.m. (n = 25–60 phagosomes). *** = P < 0.01.

Another commonly used assay for monitoring for the occurrence of cell autophagy relies on demonstrating by microscopy the transitional shift of the 18 kDa cytosolic (microtubule-associated) form of LC3 (LC3-1) to its membrane-associated, 16 kDa lipidated form, LC3-II on autophagosomes [9,10]. Using an anti-LC3 antibody, we observed, by confocal microscopy, an increased transition of the diffuse cytosolic staining pattern, typical of LC3-I, (Figure 2A; left panel) to the punctuate staining pattern, characteristic of LC3-II, within ATP-treated MDMs (Figure 2A; right panel).

### ATP-induced autophagy is dependent on mobilization of extracellular calcium

We have previously reported that ATP can stimulate Ca^2+ ^entry into cells not only through activation of ionotropic P2X purinoceptors, but also via mobilisation of intracellular Ca^2+ ^into the cytosol by activation of metabotropic P2Y receptors [7]. We then sought to establish the relative contribution of these Ca^2+ ^sources to the induction of autophagy by examining the cellular response to ATP (3 mM) stimulation for 30 minutes in both Ca^2+^-containing and Ca^2+^-free medium. In cells treated with ATP in Ca^2+^-containing media we observed the induction of autophagy by Western blot analysis as revealed by the expression of both the 16 kDa and 18 kDa forms of LC3 protein (Figure 1C). In contrast, ATP-induced cell autophagy was shown to be completely inhibited in Ca^2+^-free medium, as revealed by the absence of expression of the 16 kDa lipidated form of LC3 (LC3-II) (Figure 1C).

### ATP-induced autophagy is P2X_7 _– dependent

Having obtained preliminary evidence of P2X receptor involvement in ATP-induced cell autophagy from the above results, we sought evidence that this process involved P2X_7 _receptors. We then investigated the induction/inhibition of autophagy in response to a number of purinergic receptor agonists and antagonists. It was observed in Western blot assays (Figure 1D) that autophagy (LC3-II expression) was not induced following uridine 5'-triphosphate (UTP) (3 mM) stimulation, which activates P2Y, but not P2X receptors. In contrast, autophagy was induced by the ATP analogue, benzoylbenzoic ATP (bzATP), which selectively activates P2X_7 _receptors. To further confirm the role of the latter receptor, we pre-treated THP1 and MDM cells with either adenosine 5'- triphosphate periodate oxidized ATP (oATP); an irreversible antagonist of P2X_7_, or an anti-P2X_7 _blocking antibody, before treating with ATP. Autophagy was completely blocked in the presence of either inhibitor, as demonstrated by the absence of LC3-II expression in Western blot gels (Figure 1D), thereby confirming that ATP-induced autophagy is mediated via activation of P2X_7 _receptors.

### ATP induced phago-lysosomal fusion in infected macrophages is autophagy- dependent

We have previously reported that ATP stimulation of P2X_7 _promotes acidification of BCG-containing phagosomes within infected human macrophages [7]. Moreover, it has also been reported that induction of autophagy in RAW cells by starvation or treatment with rapamycin was associated with acidification and maturation of mycobacterial phagosomes [4]. In view of these findings, we analyzed the role of autophagy in ATP-mediated phago-lysosomal fusion within MDMs infected with green fluorescent protein expressing *M. bovis *BCG (GFP-BCG). Lysosomal trafficking was monitored by preloading BCG-infected cells for 1 hour with the acidotropic fluorescent dye Lysotracker Red that accumulates within acidic organelles [11]. The cells were then stimulated with ATP (3 mM) for 30 minutes. While control non-ATP treated cells showed minimal co-localisation of GFP-BCG with Lysotracker Red labeled lysosomes (7.2 ± 2.5%), there was significantly greater co-localisation within ATP-treated cells (63.3 ± 4.5%) (p < 0.01; student's unpaired t test) (Figures 2B &2C). The results confirmed our previously reported findings on the ability of ATP to induce phago-lysosomal fusion within BCG-infected MDMs [7]. We next examined whether pharmacological inhibitors of autophagy could block this effect. We observed that wortmannin, a classical phospatidylinositol-3 kinase (PI3 Kinase) inhibitor, which has previously been reported to inhibit the early stages of autophagosome formation [12], dramatically reduced the levels of co-localisation of BCG-containing phagosomes with lysosomes (29.5 ± 3.8%) (p < 0.01; student's unpaired t test) (Figures 2B &2C). The results support the involvement of cell autophagy in ATP-induced, phagolysosome fusion and acidification of mycobacteria-containing phagosomes. Pre-treatment of MDMs with an anti-P2X_7 _neutralising antibody also resulted in a significant reduction in the levels of co-localisation (36.8 ± 5.4%) (p < 0.01; student's unpaired t test) (Figures 2B &2C).

### Localization of mycobacteria in autophagosomes

**Figure 3:**
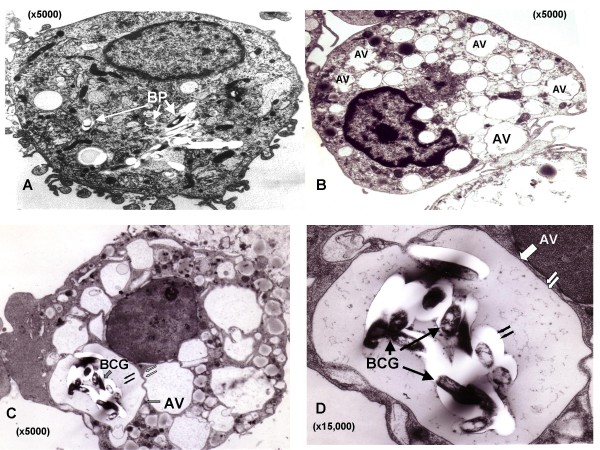
**Ultrastructural appearance of infected macrophages following exposure to ATP**. A. Electron micrograph of a non-ATP treated, BCG-infected macrophage showing the presence of the bacteria within phagosomes (BP). B. Exposure to ATP is associated with the appearance of numerous double-membrane autophagic vacuoles (AV) within the cell cytoplasm. C. A BCG-infected human macrophage (MDM) following treatment with 3 mM ATP for 30 minutes, the bacteria localize within autophagic vacuoles (AV) with clearly demonstrable inner (double black arrows) and outer (double white arrows) membranes. D. A higher magnification image of a number of mycobacteria contained within the inner and outer membranes of an autophagic vacuole.

Electron microscopic analysis of ATP-treated MDMs revealed the presence of multiple, large cytosolic autophagic vacuoles within both non-infected and BCG-infected cells (Figures 3B and 3C), while such vacuoles were almost absent in non- ATP-treated, infected cells (Figure 3A). In addition within ATP-treated infected cells, EM analysis revealed the presence of bacilli within characteristic double-membrane autophagosomes containing partially degraded internal membranes typical of maturing autophagosomes (Figure 3C &3D) [12-14].

### ATP-induced autophagy mediates rapid mycobacterial killing

**Figure 4:**
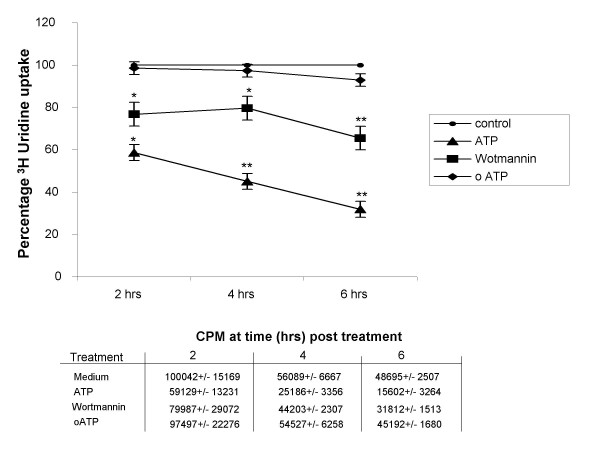
**The mycobactericidal effect of ATP is reversed by wortmannin and oATP**. MDMs, cultured for 5 days, were infected overnight with BCG (MOI of 5:1) and pulsed with ATP (3 mM) for 30 minutes, with or without pre-treatment with wortmannin (100 nM/1 hour) or oATP (0.3 mM/2 hours). The kinetics of BCG viability was determined by ^3^H-uridine incorporation, monitored at various times post treatment. The figure illustrates the pattern of results obtained from three separate experiments performed in triplicate. The symbols indicate mean values, and vertical bars indicate the standard error. The significance of the various treatment relative to the untreated control are illustrated by: ** = P < 0.005 * = P < 0.05. The results from one ^3^H-uridine uptake assay are tabulated as mean counts per minute (CPM) data +/- SEM at each time point examined.

We have previously reported that ATP induces rapid killing of intracellular mycobacteria via a cell-mediated process, as ATP had no direct bactericidal effect on BCG [6]. To examine the role of autophagy in this process, BCG-infected MDMs were pre-treated with wortmannin for 1 hr prior to pulsing with ATP (3 mM) for 30 minutes and the viability of the intracellular mycobacteria assessed at 2, 4, and 6 hours post-treatment by ^3^H-uridinine incorporation assay (Figure 4). ATP was shown to result in a marked reduction in intracellular BCG viability at 2 hr (58.6 +/- 6.35%, p < 0.05), 4 hr (45.1 ± 3.7%, P > 0.005) and 6 hrs post treatment (31.9 +/- 6.2% p < 0.005) as compared to that obtained in non-ATP-treated, infected cells, confirming previous reported findings [6]. However, pre-incubation of infected MDMs with wortmannin for 1 hr prior to exposure to ATP resulted in marked inhibition of this bactericidal effect with intracellular BCG viability at 2 hrs (76.8+/- 16.1% p < 0.05), 4 hrs (79.7 ± 5.6% p < 0.05) and 6 hrs (65.5 +/- 4.1%, p < 0.005) remaining significantly higher than in equivalent ATP-treated cells at each time point (Figure 4). To confirm that ATP was acting through P2X_7 _to induce cell autophagy, and subsequent bacterial death, infected MDMs were pre-treated with the P2X_7 _receptor antagonist, oATP, for 2 hours prior to exposure to ATP. Pre-treatment of cells with oATP was shown to result in almost the complete inhibition of ATP-induced mycobactericidal activity with intracellular BCG viability at both 4 hrs (97.4 ± 3% p < 0.005) and 6 hrs (92.9+/- 1.3% p > 0.005) post-treatment remaining similar to that obtained in non-treated cells, confirming that ATP acts via P2X_7_(Figure 4). In total the results suggest that ATP-induced mycobactericidal activity involves P2X_7 _activation and the subsequent induction of cell autophagy.

## Discussion

In this study we have shown that ATP-treated, BCG-infected THP-1 cells and MDMs undergo rapid cell autophagy, which involves the activation of P2X_7 _receptors and mobilization of extracellular calcium. Electron microscopy of ATP-treated, BCG-infected MDMs, revealed the presence of the bacteria within characteristic double-membraned autophagosomes. In addition, ATP-induced cell autophagy was accompanied by rapid phago-lysosomal fusion and loss of mycobacterial viability within infected cells suggesting that autophagy contributes to this mycobactericidal process.

Autophagy has recently been highlighted as an innate immune defense mechanism against bacterial pathogens such as Group A Streptococcus, Shigella and Mycobacterium tuberculosis [2-5]. Bacterial agents like Listeria monocytogenes and Staphylococcus aureus have also been reported to evade host immunity by subverting autophagy, thereby underscoring its importance as a potent mycobactericidal mechanism [12,13]. Earlier studies in rat hepatocytes had shown an association between reduction in the volume density of autophagic vacuoles and a fall in intracellular ATP levels [14]. In this study we sought to obtain more direct confirmation that extracellular ATP could induce autophagy in human macrophages. We demonstrated that LC3 lipidation and LC3-II association with autophagosomal membranes, which are both commonly used read-outs of autophagy [9,10], were induced by ATP, as revealed by immunoblotting and the transition of LC3 from its diffuse cytosolic appearance to a punctate intracellular distribution (Figure 1A &1B, Figure 2A).

Exploring the identity of the purinergic receptor involved in the process, we observed that ATP-induced autophagy was mediated via activation of P2X_7_, as revealed by the use of specific agonists and antagonists of this receptor. Autophagy was not induced in the absence of extracellular calcium (Fig 1c), which was consistent with the known property of P2X_7 _to promote influx of extracellular calcium into the cytosol in contrast to P2Y, which mobilize intracellular calcium.

The highly polymorphic structure of P2X_7 _has recently been described and five polymorphisms have been described that lead to reduction or loss of P2X_7 _function [15]. It has recently been shown that P2X_7 _polymorphisms were associated with impaired ATP-induced mycobacterial killing [16] and increased susceptibility to extra-pulmonary TB [8]. The present study is the first to report on the involvement of P2X_7 _in inducing cell autophagy and provides a possible rationale for the impaired mycobactericidal activity and increased predilection for extra-pulmonary TB associated with certain polymorphic forms of P2X_7_.

Our laboratory and others have previously reported that elevation of cytosolic calcium levels in ATP-treated, BCG-infected macrophages is linked to increased phago-lysosomal fusion [7,25]. Since induction of autophagy was dependent on mobilization of extracellular calcium, we explored its effect on maturation of mycobacterial phagosomes. Induction of autophagy by ATP coincided with increased acidification of mycobacterial phagosomes, which was reversed on pre-treatment of MDMs with wortmannin. In ATP-treated cells, the bacteria were found to localize within characteristic double-membraned autophagosomes. A difference in appearance and positioning of both lysostraker red labeled vesicles and GFP-BCG within wortmannin treated cells was observed as compared to anti-P2X_7 _and non-treated macrophages (Figure 2B). This was attributed to the previously reported effect of wortmannin to induce swelling of late endosomal compartments associated with PI3-K inhibition involved in membrane trafficking between late endosomes and lysosomes [26,27]. We further observed that addition of ATP had a bactericidal effect on BCG, which was reversed partially by wortmannin and almost completely by oATP (Figure 4). These findings are consistent with recent observations by Gutierrez et al who demonstrated that induction of autophagy, by starvation or rapamycin, in macrophages infected with *M. tuberculosis *(Mtb), leads to maturation of mycobacteria-containing phagosomes into phagolysosomes and suppression of mycobacterial viability [4]. Hence, induction of autophagy with ATP, as with the classical inducers like starvation or rapamycin, can override the mycobacterial block on phagosomal maturation. This ability of autophagy to induce phago-lysosomal fusion and subsequent bactericidal activity could have potential therapeutic implications. In view of the involvement of P2X_7 _receptors in this phenomenon, the development of selective, potent and safe P2X_7 _agonists as therapeutic alternatives in the treatment of multi drug resistant (MDR)-TB should be explored.

The bactericidal effect of ATP on BCG or Mtb in human and bovine MDMs has been reported previously by our laboratory and other groups [6,17,18], though the mechanism of such action has not been clearly elucidated. It has been recently shown that Mtb infection of macrophages leads to intracellular accumulation and extracellular release of ATP, which in turn activates P2X_7 _receptors to mediate cellular apoptosis and bacterial killing [19]. Apoptosis was induced in this latter study in 26.5 ± 2.5% of macrophages in response to stimulation with 5 mM ATP at an MOI of 10:1, following 48 hours of infection [19]. However, the evidence for ATP-induced apoptosis is still equivocal, as we have found that caspase inhibitors do not block ATP-mediated killing of intracellular mycobacteria although they do block IL-1β release [20]. Moreover, in contrast to ATP, classical inducers of cell apoptosis such as FasL or anti-Fas antibody are relatively ineffective at reducing intracellular BCG viability within infected human macrophages [6,21]. In addition, using a lower dose of ATP (3 mM), a lower MOI (5:1) and a lower duration of culture, we failed to observe any difference in apoptosis levels between ATP-treated and control cells, as revealed by flow cytometric determination of active caspase-3 expression (data not shown). Though it is probable that macrophage apoptosis plays a role in ATP-mediated mycobacterial killing, the rapid decline in intracellular BCG viability immediately after ATP exposure appears to be primarily mediated by autophagy. This is consistent with our observation that wortmannin, a classical inhibitor of autophagy, achieved only partial reversal of bactericidal activity while the P2X_7_-antagonist, oATP, almost completely inhibited ATP-induced bacterial killing (Figure 4). Similarly, in our confocal studies neither anti-P2X_7 _or wortmannin treatment completely ablated the co-localisation observed following ATP stimulation (Figure 2B &2C), implying that other mechanisms are involved in mediating this effect. The results suggest that ATP-induced mycobactericidal activity is dependent on both autophagy as well as apoptosis. The use of a more specific P2X_7 _inhibitor such as KN62 or combinations of inhibitors such as anti-P2X_7 _and wortmannin, which may exhibit synergistic activity may help to clarify the relative roles of each process. That autophagy is involved in ATP-mediated mycobactericidal activity is supported by previous reports describing profound changes in the architecture of the intracellular vacuolar system of ATP-treated infected macrophages [[17], our unpublished observations]. However, these changes were also accompanied by membrane blebbing and nuclear condensation (results not shown), which are morphological changes associated with early stages of apoptosis supporting a role for both processes in the associated mycobactericidal effect. Multiple, large cytosolic vacuoles were also formed within non-BCG infected cells following ATP treatment (Figure 3B). Although no detailed assessment was made of their structure in terms of whether they represented autophagosome formation, it was noted that cellular debris was apparent in a high percentage of them. Our laboratory is currently undertaking a detailed assessment of such structures to determine the degree of autophagosome formation within ATP-treated macrophages.

That autophagy may prove a critical mycobactericidal effector mechanism utilized by phagocytes is also suggested by the fact that we have previously reported, using macrophages derived from p47Phox-/- and iNOS-/- mice, that neither oxygen or nitrogen radical generation are involved in mediating ATP-induced, mycobactericidal activity [22]. Moreover, parallel studies performed in BCG-infected macrophages, derived from both NRAMP resistant and susceptible strains of mice, also failed to reveal any influence of this additional macrophage-associated, anti-bacterial mechanism on the mycobactericidal effects of ATP [22]. Thus many of the classical, cell-mediated, antibacterial effector mechanisms of oxygen and nitrogen radical generation, or Nramp protein expression do not appear to be involved in, and hence as effective as induction of autophagy in the ATP-mediated killing of intracellular mycobacteria.

Work is currently underway in our laboratory to further analyze the relative roles played by autophagy and apoptosis and their inter-dependence in the mycobactericidal activity mediated by ATP.

## Conclusion

We report that exposure to ATP induces human monocytes/macrophages to undergo rapid cell autophagy, through a P2X_7_-dependent mechanism. Induction of autophagy is associated with augmented mycobactericidal activity of macrophages, which is mediated through increased phago-lysosomal fusion of mycobacterial phagosomes. These results support the importance of autophagy as an innate, cellular, mycobactericidal mechanism and highlight a potential therapeutic role for P2X_7 _agonists in the treatment of MDR-TB.

## Methods

### Reagents

ATP, bzATP, oATP, UTP, wortmannin, ethylene glycol-bis(2-aminoethylether)-N,N,N',N'-tetraacetic acid (EGTA) and calcium chloride (CaCl_2_) were purchased from Sigma (Poole, UK). The rabbit anti-LC3 polyclonal antibody and anti-rabbit secondary antibody were obtained from Novus Biologicals and Pierce Biotechnology respectively. The anti-P2X_7 _antibody was a kind gift from Dr Gary Buell (Geneva Biomedical Research Institute). Lysotracker Red was purchased from Cambrex Biosciences.

### Cells and bacteria

Peripheral blood mononuclear cells (PBMC) were isolated from EDTA blood taken from healthy individuals by standard Ficoll-Hypaque (GE Healthcare Biosciences) gradient separation. Monocytes were then isolated from the PBMC by overnight adherence to 75 ml plastic tissue culture flasks (Corning), washed thrice to remove any non-adherent cells and resuspended by chilling on ice for 30 mins and pipetting with pre-chilled (4°C) PBS. The monocytes were washed and re-suspended (2.5 × 10^6 ^cells/ml) in RPMI 1640 medium (Life Technologies, Paisley, U.K.) containing 5% pooled AB^+ ^male human serum (First Link, West Midlands, U.K.), 2 mM L-glutamine (Life Technologies) (complete medium) and rh-GM-CSF (90 IU/ml) (Berlex USA).

THP-1 cells were obtained from the American Type Culture Collection (Manassas, VA) and cultured in RPMI 1640 medium + 10% fetal calf serum + 2 mM L-glutamine. The cells were used in a non-activated/differentiated state to maintain their early monocytic phenotype.

For experiments using calcium-free media, the cells were grown in standard Hank's Balanced Salt Solution (HBSS) or in calcium free HBSS + 2 mM EGTA. The latter was added to chelate any residual Ca^2+^.

Stock cultures of *M bovis*-BCG (Evans strain) were maintained in log phase growth in Middlebrook 7H9 broth (Difco, Detroit, MI) supplemented with 10% Middlebrook ADC enrichment media (Difco) and 0.02% Tween (Difco) at 37°C. The concentration of the BCG stock was determined by direct counting using a Thoma counting chamber (Weber Scientific UK) under dark ground illumination.

GFP-BCG was obtained as a kind gift from Prof D. Young (Imperial College of London, London, U.K). The BCG contained the gene encoding a FACS-optimized GFP protein [23] constitutively expressed under the control of the mycobacterial heat shock protein 60 promoter in a pSMT3 shuttle vector construct [24]. Stock aliquots, stored in glycerol at -70°C, were grown to log phase in 7H9 broth supplemented with 10% ADC enrichment medium, 0.2% Tween 80, and 50 μg/ml hygromycin (Sigma, St. Louis, MO).

### Western blot assay

Following exposure for 30 min to 3 mM ATP, THP1 cells and blood-derived monocytes were washed once in PBS and resuspended in RIPA buffer + protease inhibitor (Sigma, St. Louis, MO) and the cell lysate stored at -80°C prior to assay. SDS-PAGE was performed using a 15% discontinuous gel at 120 V. Following electrophoresis the resolved proteins were transferred onto a PVDF membrane at 80 V for 60 minutes, using a Biorad transfer apparatus. The blot was removed from the transfer apparatus and blocked overnight at 4°C in TBS-T, containing 5% nonfat dried milk. The blot was washed thrice in TBS-T following overnight incubation and then probed with a rabbit polyclonal anti-LC3 antibody (diluted 1:1000 in TBS-T/milk) for 60 minutes. After 3 rinses in TBS-T, peroxidase-conjugated anti-rabbit secondary antibody was applied for 1 hour and the excess antibody removed by washing thrice in TBS-T and the reaction developed using Supersignal West Pico ECL reagents.

In experiments incorporating purinergic receptor agonist and antagonists, oATP (0.3 mM) or anti-P2X_7 _antibody (3 μg/ml) were added to the cells for 2 hr/37°C and 1 hr/37°C respectively, prior to addition of ATP (3 mM) for 30 minutes/37°C. UTP (3 mM), and bzATP (3 mM) were similarly added to cells for 30 minutes/37°C before processing them for western blot analysis for LC3 protein expression.

### Confocal fluorescent microscopy

MDMs were grown to sub-confluence on poly-L-lysine coated glass-bottom dishes (MatTek) at approximately 5 × 10^5 ^cells/dish for 5 days. GFP-BCG were then added to the macrophages at an MOI of 5:1 and incubated overnight. Following removal of extracellular bacteria by multiple washing, LysoTracker red (75 nM) was added to the cells for 1 hour at 37°C to stain lysosomes. Wortmannin (100 nM) and anti-P2X7 antibody (3 μg/ml) was then added for 1 hr at 37°C before addition of ATP (3 mM) in appropriate wells. Following exposure to ATP for 30 minutes, the cells were washed in RPMI. Cells were imaged live using a Zeiss LSM510 confocal microscope using excitation wavelengths of 488 and 546 nm to visualize GFP and Lysotracker Red respectively. For LC3 staining, MDMs were grown on poly-L-lysine coated glass coverslips for 5 days. Following treatment with ATP, cells were fixed with methanol at -20°C for 30 minutes. Cells were then washed with PBS and incubated in PBS containing 5% donkey serum to block non-specific binding. Cells were incubated with anti- LC3 antibody (1:500) for 1 hour, followed by three washes in PBS and treatment with anti-rabbit secondary antibody, conjugated with AlexaFluor 555 (Invitrogen), for 1 hour. Cells were washed 5 times in PBS and mounted onto slides with Vectashield (Vector Laboratories, CA) as a mounting medium. Fluorescence was visualized by confocal microscopy.

### Electron microscopy

Cells were prepared for transmission electron microscopy (TEM) by pelleting the various cell preparations in 1.5 ml eppendorfs following centrifugation for 1 min/6000 rpm in an eppendorf (Hettich) centrifuge. The cell pellets were then fixed in freshly prepared gluteraldhyde fixative and stored at 4°C until ready for processing. TEM processing of the cell samples was performed at the EM microscopy suite, University of Birmingham according to standard fixation protocols.

### BCG viability assay

Macrophages were grown to sub-confluence (approx 5 × 10^4^cells/well) in 96-well round-bottom microtiter plates (Corning) at 200 μl/well. A total of 2.5 × 10^5 ^BCG per well was added (MOI 5:1) and incubated at 37°C/5%CO_2 _overnight. Excess BCG was removed by washing thrice in RPMI. Where appropriate, wortmannin (100 nM) and oATP (0.3 mM) were added 1 and 2 hr respectively prior to addition of ATP (3 mM). Intracellular BCG viability following exposure of infected cells to ATP for 30 min, was assessed by ^3^H-uridine incorporation assay as this provided a much more rapid assessment (within 72 hrs) of the mycobacterstatic effect of ATP as compared to colony forming unit (CFU) assay assessment which takes > 14 days to form countable colonies. The BCG-infected cells were washed and cultured in RPMI +5%AB^+ ^serum and at appropriate time points, a total of 150 μl cell supernatant was removed and transferred to a separate 96 well plate. Cell lysis was performed on the remaining cell pellets by addition of 50 ul of a 0.2% saponin solution for 30 min/37°C. After 30 min, 100 μl of Middlebrook 7H9 (+10% ADC supplement) was added to each well of both the cell supernatant and pellet lysates followed by addition of 20 μl/well of a 100 μCi/ml stock of ^3^H-uridine (Amersham UK). The wells were harvested after 72 hours on a '1205 betaplate scintillation counter' (Wallac) and the combined counts/min of the matched cell supernatant and pellet wells were taken as an indicator of relative bacterial viability.

### Statistics

The Student's *t*-test was used for all statistical comparisons.

## Authors' contributions

DB carried out the majority of the experiments. OSQ performed the confocal microscopy studies, W–YL performed the flow cytometry studies analysing active caspase 3 expression, DAL performed the electron microscopy studies. Experimental design and manuscript preparation were carried out by DB and DAL. All authors have read and approved the manuscript.
